# Ovarian function and reproductive outcome after ovarian tissue transplantation: a systematic review

**DOI:** 10.1186/s12967-019-02149-2

**Published:** 2019-11-29

**Authors:** Sepideh Sheshpari, Mahnaz Shahnazi, Halimeh Mobarak, Shahin Ahmadian, Alberto Miranda Bedate, Ziba Nariman-Saleh-Fam, Mohammad Nouri, Reza Rahbarghazi, Mahdi Mahdipour

**Affiliations:** 1 Department of Midwifery, Faculty of Nursing and Midwifery, Tabriz University of Medical Sciences, Tabriz, 5166615739 Iran; 2 Department of Clinical Sciences, Faculty of Veterinary Medicine, Ferdowsi University of Mashhad, Mashhad, 9177948974 Iran; 3 Department of Biology, Faculty of Science, Azerbaijan Shahid Madani University, Tabriz, 537517169 Iran; 4 Laboratory for Translational Immunology (LTI), Universitair Medisch Centrum Utrecht, (UMCU), Heidelberglaan 100, 3584 CX Utrecht, The Netherlands; 5 Women’s Reproductive Health Research Center, Tabriz University of Medical Sciences, Tabriz, 5166615739 Iran; 6 Department of Applied Cell Sciences, Faculty of Advanced Medical Sciences, Tabriz University of Medical Sciences, Tabriz, 5166615739 Iran; 7 Stem Cell Research Center, Tabriz University of Medical Sciences, Tabriz, 5166615739 Iran; 8 Department of Reproductive Biology, Faculty of Advanced Medical Sciences, Tabriz University of Medical Sciences, Daneshgah St., Tabriz, 5166615739 Iran

**Keywords:** Ovarian tissue, Transplantation, Cryopreservation, Fertility, Pregnancy

## Abstract

The aim of this systematic review study is to summarize the current knowledge of ovarian tissue transplantation and provide insight on ovarian function, fertility and reproductive outcome following ovarian tissue transplantation. Relevant studies were identified by searching through PubMed, Cochrane Library, Embase, ProQuest, and Scopus databases until August 2018. Ovarian function by examination of the hormonal level was evaluated, together with follicular growth, the return of menstrual cycle and assessment of reproductive consequences: pregnancy, miscarriage rates and live birth after transplantation. Studies including female patients aged between 22 and 49 years that were subjected to ovarian tissue transplantation were considered. A total of 1185 studies were identified in the primary search. Titles and abstracts were screened for assessment of the inclusion criteria. Finally, twenty-five articles met the criteria and were included in this study. In general, 70% of patients that underwent ovarian tissue transplantation had ovarian and endocrine function restoration as well as follicular growth. Pregnancy was reported with 52% of the patients. The available evidence suggests that ovarian tissue transplantation is a useful and an applied approach to restore hormonal function, endocrine balance and eventually fertility outcomes in patients that are predisposed to lose their fertility, diagnosed with premature ovarian failure (POF), as well as women undergoing cancer treatments. Identification of the techniques with the lowest invasions for follicular and oocyte development after ovarian tissue transplantation aiming to reduce probable adverse effects after treatment is indispensable.

## Introduction

In the past decades, the life expectancy of patients diagnosed with most forms of cancers has increased due to improved and novel therapeutics [1], highlighting the importance of quality of life after treatment. Besides the increased efficiency of anti-cancer treatments such as chemotherapy and radiotherapy, there are also negative side effects, especially on the reproductive system that affect fertility [2, 3]. Following the use of alkylating agents and ionizing radiation in cancer treatment, endocrine activity and ovarian function can be severely compromised. A profound reduction in ovarian follicle numbers coincides with ovarian damage following chemotherapy, and close relationships exist between patient’s age, drug dosage and risk of losing ovarian function [2]. Chemotherapy, particularly when alkylating agents are used, can lead to premature ovarian failure (POF) as one of the complications [3]. POF is known as an ovarian function insufficiency affecting about 1% of women before the age of 40 [4], whereas over one-third of women undergoing chemotherapy procedures suffer from POF [5]. Male cancer patients are not exempt from post-treatment complications and also require attention to assist the return of their reproductive function [2].

**Fig. 1:**
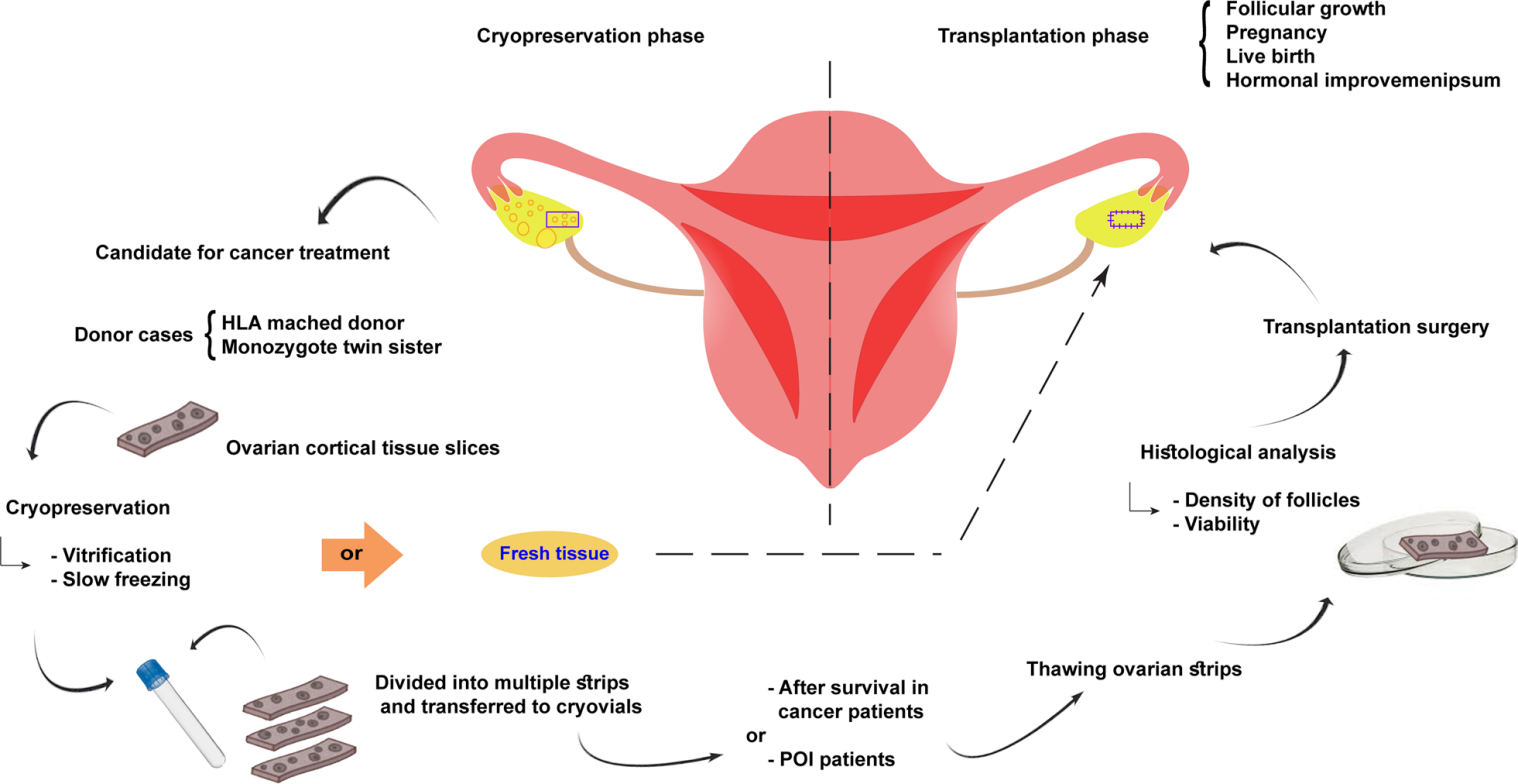
Schematic presentation of ovarian tissue cryopreservation and transplantation

Various assisted reproductive techniques are being offered for female patients before and after therapy such as oophoropexy for radiation shielding, fertility-sparing surgery, egg and embryo freezing, ovarian tissue cryopreservation and transplantation [6]. Despite the fertility preservation options present today, in some cases it is important to begin treatment without any delay. In addition, in prepubertal patients, ovarian stimulation to obtain oocytes is not considered. For those patients it can be beneficial to remove the ovarian tissue via laparoscopy followed by tissue cryopreservation. When the patients are recovered the preserved tissue could be transplanted to its original or heterotopic site [7]. Ovarian tissue transplantation can restore endocrine function and fertility in women with premature ovarian insufficiency [8]. The first successful fresh ovarian tissue transplantation leading to living birth in primates was reported in 2004 [9]. In another study, Donnez et al. transplanted ovarian tissue 6 years after the diagnosis of Hodgkin’s lymphoma. An improved ovarian function was observed 5 months after transplantation and was followed by a live birth [7]. About type of transplant, in auto-transplantation, fresh or cryopreserved ovarian tissue is transplanted to the same person, or to identical twin sister, [10] whereas in allotransplantation the patient receives tissue from a genetically different HLA matched-donor. Both fresh and frozen ovarian tissue transplantations have been reported to yield similar outcomes considering menstrual cycle return and ovarian function restoration [11]. In Fig. 1, the processes of cryopreservation and transplantation are summarized. Vitrification and slow freezing are two common techniques applied for ovarian tissue cryopreservation. In slow freezing technique, low level of follicular degradation and higher degree of tissue survival and follicular counts were notified, however, no significant differences were reported between these techniques [12, 13]. This study makes an inventory and summarizes the current knowledge of ovarian tissue transplantation and its success or failure to maintain fertility, restore reproductive capacity and preserve ovarian function. The current systematic review evaluates the success rate of ovarian transplantation and proposes a guideline for fertility preservation in the future.

## Methods

The paper was prepared based on the standards and guidelines of the Preferred Reporting Items for Systematic Reviews (PRISMA) [14]. All data originate from previously published experiments in international peer-reviewed journals.

### Search strategy, data extraction, and eligibility criteria

Relevant studies were identified by searching through PubMed, Cochrane Library, Embase, ProQuest, and Scopus databases until August 2018 with the following keywords: “ovarian tissue transplantation”, “ovary transplantation”, “ovarian implantation”, “ovary implantation”, “ovarian tissue allografting”, “ovarian auto-transplantation”, “ovary allografting”, “birth rate”, “live birth”, “pregnancy”, “pregnancy rate”, “chemical pregnancy”, “clinical pregnancy”, “AMH”, “anti Mullerian hormone”, “follicular stimulating hormone”, “FSH”, “atrial follicle count”, “fertilization”, “fertility rate”, “reproductive capacity”, and “treatment of infertility” in human studies. References were collected in EndNote X7.1 (Thomson Reuters, USA).

### Study selection

After the primary search, title and abstract of studies were first evaluated by three authors of this paper (H.M, S.SH, and SH.A). If the abstracts fulfilled the general aspect of the review, the full text was considered. Inclusion criteria such as written language (English), article type (original article), intervention (ovarian tissue transplant) and outcome (hormone and ovarian tissue function and fertility) were regarded to select the full text of the remaining articles in the study. The term transposition instead of transplantation, non-English language and cryopreservation of ovarian tissue without transplantation were used as the exclusion criteria. Authors independently reviewed the selected published articles. The eligible studies had the methodological characteristics such as type of the study, number of participants and their disease condition, type of interventions, number of patients that underwent ovarian tissue transplantation, age at the time of intervention, outcomes and an approximate follow-up duration in the post-intervention period. Any disagreements that arose between the reviewers were resolved through discussion, or with a fourth reviewer (M. M). Relevant data were summarized in a tabular format in a systematic manner (Table 1).

**Table 1:** Summary of the results from 29 included articles identified in a systematic review of the literature

Study design	Participants underwent ovarian tissue transplantation	Intervention	Time of procedure	Follow up (median range)	Outcome(s)	(Author, year)
Case report	A woman with diagnose of anal cancer	Auto-transplantation/frozen tissue	30.5 years	225 days	Ovarian function by follicle growth and FSH, LH and E2	Dittrich et al. 2008 [15]
Technical note	A woman with diagnose of Hodgkin disease and relapse	Auto-transplantation/frozen tissue	33 years	Not reported	Ovarian function, fertility and pregnancy	Dittrich et al. 2012 [16]
Retrospective analysis	11 women with hematological malignancy, four with Brest cancer, tree with anal cancer and two with ovarian cancer	Auto-transplantation/frozen tissue	Mean age 34.2 years	Ongoing to date of study	Ovarian function and pregnancy	Dittrich et al. 2015 [17]
Case report	A woman with diagnose of Hodgkin’s lymphoma	Auto-transplantation/frozen tissue	32 years	1 year	Ovarian activity with FSH, E2 and LH, fertility	Muller et al. 2012 [18]
Case report	A woman with diagnose of primary Sjogren’s syndrome	Auto-transplantation/frozen tissue	42 years	244 days	Follicular development	Wølner-Hanssen et al. 2005 [19]
Case report	A woman with diagnose of invasive ductal breast carcinoma	Auto-transplantation/frozen tissue	44 years	7 months	Ovarian function by hormonal assay and follicular development, pregnancy	Burmeister et al. 2013 [20]
Case report	A woman with diagnose of b-thalassemia	Auto-transplantation/frozen tissue	29 years	12 months	Restoration of ovarian function, pregnancy, and live birth	Revelli et al. 2013 [21]
Case report	A woman with papillary thyroid carcinoma and metastases of neck lymph node	Auto-transplantation/frozen tissue	26 years	2 years	Ovarian function by FSH, LH, E2 and AMH and pregnancy	Kiseleva et al. 2015 [22]
Case report	A woman with diagnose of sickle-cell anemia	Auto-transplantation/frozen tissue	27 years	4 years	Ovarian function by hormonal level, follicular development and menstrual cycle, pregnancy and live birth	Demeestere et al. 2015 [10]
Case report	A woman with diagnose of Ewing’s sarcoma	Auto-transplantation/frozen tissue	28 years	5 years	Pregnancy and live birth	Ernst et al. 2010 [23]
Case report	A woman with Hodgkin lymphoma	Auto-transplantation/frozen tissue	30.5 years	5 years	Restoration of ovarian function, fertility and pregnancy outcome	Oktay et al. 2011 [24]
Prospective cohort	A woman with thalassemia	Auto-transplantation/frozen tissue	29 years	6.5 years	Gonadal function by FSH, AMH and E2 levels	Biasin et al. 2015 [25]
Prospective cohort	Four women aged 46–49 year with abdominal hysterectomy and bilateral salpingo oophorectomy for uterine leiomyomas	Auto-transplantation/fresh and frozen tissue	46–49 years	1 year	Ovarian function by serum E2 and FSH and follicular growth	Callejo et al. 2001 [26]
Case report	Three patient of 20, 15 and 12-year old women with diagnose of β-thalassemia major, homozygous sickle cell anemia and AML, respectively underwent chemotherapy and TBI before BMT	Allo-transplantation/fresh tissue	Respectively 35, 32, 32	1 year	Ovarian function by hormonal level and follicular development	Donnez et al. 2010 [27]
Prospective cohort	Three women were included; a 23-years old woman with colorectal cancer dukes b2, 34 years old woman with infiltrating lobular carcinoma and 29 years old woman with Hodgkin’s lymphoma, mixed-cellularity sub-type	Auto-transplantation/frozen tissue	31, 41 and 39 years	91 Weeks	Ovarian function by hormonal assessment, ultrasound examination and follicular development	Fabbri et al. 2014 [28]
Prospective cohort	10 women with Hodgkin’s lymphoma, sickle cell anemia, Hodgkin’s lymphoma, non-Hodgkin lymphoma, Wegener’s granulomatosis, cerebral tumor, tubo-ovarian abscess, endometriosis, major-thalassemia, AML, sickle cell anemia	Auto and allo-transplantation/fresh and frozen tissue	24–35 years	2.5 years	Ovarian function analyzed by systemic levels of FSH, LH, inhibin B, E2, and AMH	Janse et al. 2011 [29]
Retrospective cohort	41 women with breast cancer, Mb. Hodgkin, non-Hodgkin, cervical cancer, aplastic anaemia, Ewing sarcoma, paroxysmal nocturnal, Haemoglobinuria sarcoma, Haemolytic uraemic syndrome, ovarian cancer, colon cancer, anal cancer and others	Auto-transplantation/frozen tissue	Mean age: 32.9 years	10 years	Ovarian function and fertility outcome	Jensen et al. 2015 [30]
Prospective cohort	Five women presented with cervical cancer (3), breast cancer (1), and Hodgkin’s lymphoma (1)	Auto-transplantation/frozen tissue	Mean age 31	7 years	Ovarian function by (FSH, LH, estradiol, progesterone, and testosterone, follicular growth by ultrasound	Kim 2012 [31]
Retrospective cohort	A woman at the age of 28 with B cell non-Hodgkin’s lymphoma	Auto-transplantation/frozen tissue	One 31-year-old patient	30 months	Ovarian activity by hormonal assay and follicular growth	Meirow et al. 2007 [32]
Case report	A woman with mature cystic teratoma and underwent right salpingo-oophorectomy	Auto-transplantation/frozen tissue	34 years old	4 years ongoing	Ovarian function by hormonal level and Doppler ultrasound	Fabbri et al. 2018 [33]
Prospective cohort	20 cancer survivors’ women with CML, Hodgkin’s, Breast cancer, non-Hodgkin’s lymphoma, Ewing’s sarcoma and AML developed into POF	Auto-transplantation/frozen tissue	22–45 years	7 to 141 months	Endocrine profile, IVF, pregnancies, live births	Meirow et al. 2016 [34]
Prospective cohort	10 pairs of identical twin sisters out of which one sister of each pair diagnosed with POF received ovarian transplants from other normal sister also another woman whom received her own cryopreserved ovarian tissue at the time of transplantation	Auto and Allo-transplantation/fresh and frozen tissue	24–40 years	5 years	Menstrual cycle, hormone levels, pregnancy, live birth, duration of transplant function, and ovarian tissue evaluation	Silber et al. 2010 [35]
Prospective cohort	17 infertile women with POI	Auto-transplantation/frozen tissue	ND	Not reported	Follicle growth, serum estrogen level and IVF-embryo transfer	Suzuki et al. 2015 [36]
Retrospective cohort	Two patients with T cell lymphoma and Hodgkin lymphoma	Auto-transplantation/frozen tissue	24 and 22 years	40 months	Ovarian function by FSH and AMH and pregnancy	Tanbo et al. 2015 [37]
Retrospective cohort	38 women with hematologic neoplasia (17); with breast carcinoma (10); borderline ovarian cancer and tumor (4), anal cancer (3); premature ovarian failure (2); ovarian cancer (1) and cervical cancer (1)	Auto-transplantation/frozen tissue	27–44 years	8 years	Ovarian function confirmed by hormonal analysis, onset of menstrual cycle or elevation of systemic levels of estradiol	Beckmann et al. 2017 [38]
Case report	One 24 years old patient with diagnose of Hodgkin’s disease stage iv	Auto-transplantation/frozen tissue	29 years	267 days	FSH, LH, Follicle count and pregnancy	Demeestere et al. 2006 [39]
Retrospective cohort	Eight women diagnosed with cancer followed by POF underwent ovarian tissue transplantation for fertility	Auto-transplantation/frozen tissue	22–39 years	7 years	Ovarian function and fertility	Imbert et al. 2014 [40]
Case report	A woman at the age of 18 with acute severe pelvic pain	Auto-transplantation/frozen tissue	28 years	8 months	Serum estradiol and FSH, follicular development and pregnancy outcome	Povoa et al. 2016 [41]
Prospective cohort	22 patients include 11 POF and 11 cancer patients with Hodgkins, brain cancer, MS, Blood Disorder, Synovial sarcoma and breast cancer	Auto and allo-transplantation/fresh and frozen tissue	ND	In 19 with over 1-year follow-up and in other 240 days	Ovarian function by FSH, LH, estradiol, AMH, and menstrual cycle	Silber et al. 2015 [11]

*AML* acute myeloid leukemia *ALL* acute lymphoid leukemia *CML* chronic myeloid leukemia *TBI* total body irradiation *BMT* bone marrow transplantation, *POF* premature ovarian failure, *POI* premature ovarian insufficiency, *HSCT* hematopoietic stem cell transplantation, *FSH* follicle stimulating hormone, *LH* Luteinizing hormone; *AMH* anti-Mullerian hormone *E2* estradiol *ND* not determined

### Outcomes measures

The primary outcome of this systematic review was to assess systemic levels of follicle stimulating hormone (FSH), Luteinizing hormone (LH), anti Mullerian hormone (AMH), and inhibin as well as follicular growth, and the return of a menstrual cycle after ovarian tissue transplantation. Pregnancy rates, live births, and in vitro fertilization (IVF) consequences were considered as secondary outcomes.

## Results

**Fig. 2:**
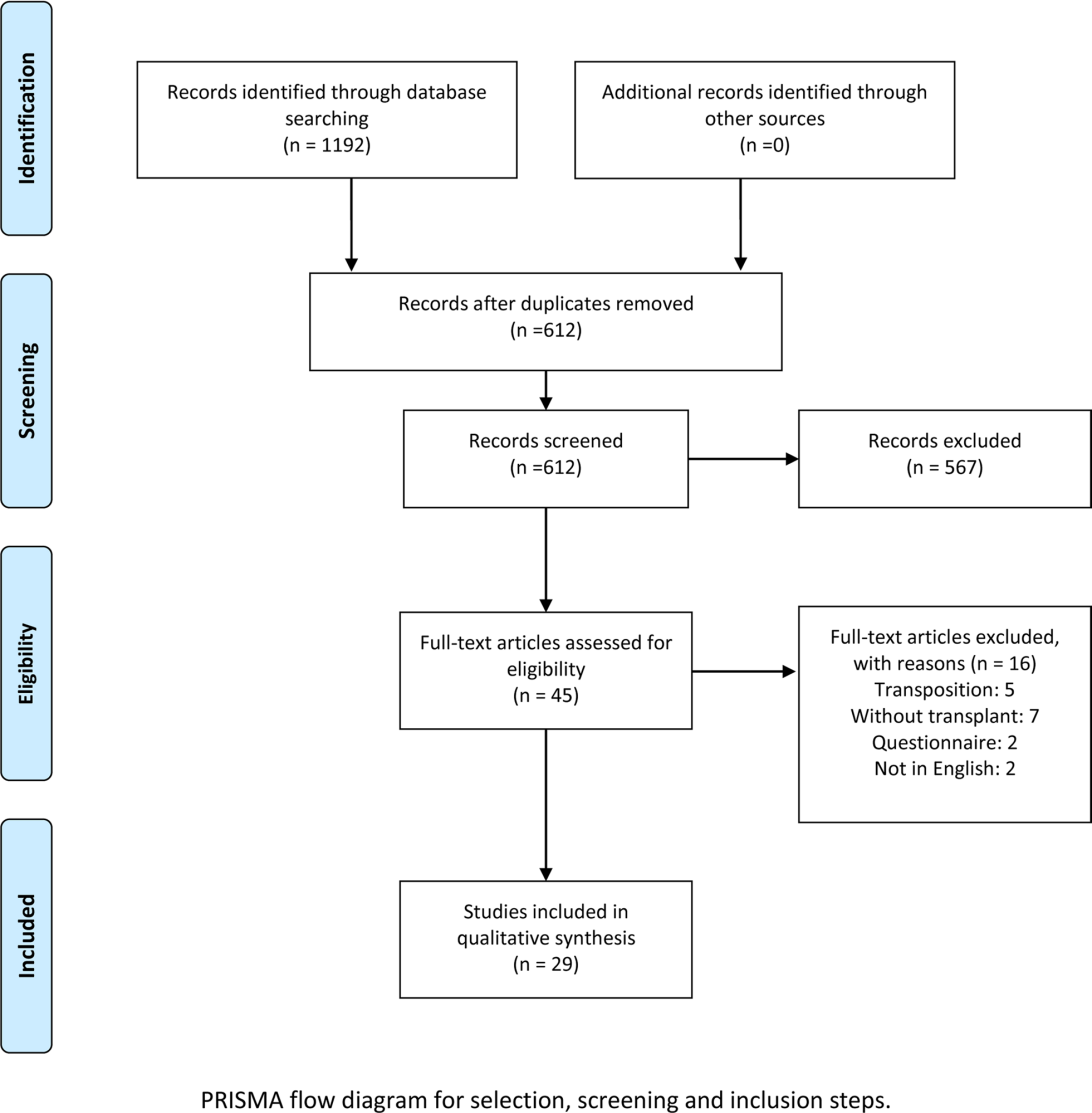
PRISMA flow-chart indicated the eligible studies selection and screening process

A total of 1192 studies were identified in the primary search (Fig. 2). Among these studies, titles and abstracts were carefully reviewed by independent reviewers assessing whether they could meet the Eligibility Criteria. At this stage, 612 studies were excluded since they did not match the inclusion criteria of the study. Finally, 29 unique reports, including cohort articles and case reports, were enrolled in this study. The selected articles were critically appraised by reviewers for methodological quality.

### Clinical characteristics of the selected studies

In the included studies, a total of 693 patients underwent ovarian cryopreservation whereas only 210 patients were subjected to ovarian tissue transplantation. Among transplant recipients, 29 patients received fresh ovarian tissue, out of which 26 received transplants from their twin sisters or HLA-matched donors and 3 cases received their own ovaries; 11 patients received allogenic frozen tissue, and 170 received their own frozen ovarian tissue. The mean age for transplantation was 31 years; with the youngest patient being a 22 years old women cured from Hodgkin lymphoma, without children. The oldest patient in the included manuscripts was 49 years old and ovarian tissue was grafted ectopically in her arm to prevent the occurrence of menopause [26].

The most common reason for ovarian tissue transplantation was fertility preservation cancer treatment (165 cases), followed by women suffering from POF (40 cases). One patient diagnosed with a hemi-uterus had her fallopian tubes removed by laparotomy [41]. In 4 patients diagnosed with uterine leiomyoma and candidate for ovarian cancer, abdominal hysterectomy was performed with bilateral salpingo-oophorectomy and subsequently, the ovarian tissues were cryopreserved [26].

Various sites have been used for ovarian transplantation such as the abdominal rectus muscle, peritoneum, abdominal wall, adjacent ligament of the ovary, ovarian cavity and fallopian tube (Table 2). Only in one study, the transplant group was compared with ten peri-menopausal patients as a control group for hormonal evaluation [26]. Some subjects were excluded from the studies since they either had less follow-up duration or were lost to follow-up [30, 34, 37, 40].

**Table 2:** Summary of primary outcomes from selected studies

Total number of patients in study	Second transplantation	Third transplantation	Transplantation site	Number of patient with therapeutic outcome	Onset of ovarian function	Duration of Ovarian function	FSH below 20 (IU/l)	E2 above 100 (pg/ml)	Follicular growth	(Author, year)
1	1	0	Right pelvic side wall in peritoneal pocket	1	2.5 months	Not reported	1	1	1	Dittrich et al. 2008 [15]
1	0	0	Pouch of peritoneum in the Board ligament	1	3 months	Not reported	1	1	1	Dittrich et al. 2012 [16]
20	0	0	Peritoneal pocket, below the fallopian tube, Pelvic wall, Ovary	19	4 months	average 1.7 years and Ongoing in 13 patient to date of study	ND	19	ND	Dittrich et al. 2015 [17]
1	0	0	Ovarian fossa of the right pelvic wall	1	3 months	Not reported	1	1	1	Muller et al. 2012 [18]
1	0	0	Right forearm	1	Not reported	7 months	ND	1	1	Wølner-Hanssen et al. 2005 [19]
1	0	0	Ovary	1	4 months	5 months ongoing	1	ND	1	Burmeister et al. 2013 [20]
1	0	0	Ovary	1	3 months	12 months	1	ND	1	Revelli et al. 2013 [21]
1	0	0	Ovary	1	6 months	2 years ongoing	ND	ND	1	Kiseleva et al. 2015 [22]
1	0	0	Residual left ovary, right peritoneal bursa and trocar incision	1	4 months	4 years	1	1	1	Demeestere et al. 2015 [10]
1	0	0	Ovary	1	Not reported	4 years ongoing	ND	ND	1	Ernst et al. 2010 [23]
1	0	0	Lower abdominal wall	1	2 months	5 years	1	ND	1	Oktay et al. 2011 [24]
47	0	0	Orthotopic site	1	3 months	Not reported	ND	ND	ND	Biasin et al. 2015 [25]
14	0	0	Arm (SC), rectus abdominis muscle	3	2–4 months	Not reported	3	3	3	Callejo et al. 2001 [26]
3	0	0	Ovarian cortex	3	3.5–7 months	1 years	3	3	3	Donnez et al. 2010 [27]
3	0	0	Subcutaneous pockets	3	2–4 months	91, 61 and 76 weeks ongoing	3	3	3	Fabbri et al. 2014 [28]
10	2	0	Peritoneal incision close to the ovary or on decorticated ovaries	9	3.5–7 months	9–86 months 17–28 months	6	5	ND	Janse et al. 2011 [29]
41	11	1	Remaining ovary and into a peritoneal pocket	40	Not reported	Not reported	40	ND	ND	Jensen et al. 2015 [30]
5	4	0	Heterotopic site such as rectus muscle and sheath	5	2–4 months	9–84 months	1	1	1	Kim et al. 2012 [31]
56	0	0	Ovary	1	Not reported	30 months	ND	ND	1	Meirow et al. 2007 [32]
1	1	0	Peritoneal pocket, left ovary	ND	Not reported	Not reported	ND	ND	1	Fabbri et al. 2018 [33]
20	0	0	Subcortical tunnels and peritoneum in the ovarian ligaments	19	Not reported	Not reported	12	11	ND	Meirow et al. 2016 [34]
25	0	0	Denuded medulla	22	4.5 months	All more than 2 years, ½ 6 years, 2 for 8 years	22	ND	ND	Silber et al. 2010 [35]
47	0	0	Beneath the serosa of fallopian tubes	ND	Not reported	Not reported	ND	ND	9	Suzuki et al. 2015 [36]
164	0	0	Remaining ovary	2	Not reported	40 months	ND	ND	ND	Tanbo et al. 2015 [37]
399	5	1	Peritoneal pocket	19	1 years	Not reported	ND	ND	ND	Beckmann et al. 2017 [38]
1	0	0	Three fragments were placed into the incision in the ovary and nine into the peritoneal pocket	1	3 months	Not reported	1	1	1	Demeestere et al. 2006 [39]
225	1	0	Peritoneal and ovarian sites and orthotopic site. Other peritoneal sites including ovarian bursa and subcutaneous abdominal trocar incision	4	2–5 months	1–5 years 3 cases ongoing	4	N	N	Imbert et al. 2014 [40]
1	0	0	Ovarian fossa and broad ligament	1	Not reported	Not reported	1	1	1	Povoa et al. 2016 [41]
22	2	2	Peritoneum of the denuded fallopian tube isthmus	11	60–130 days	3–4 years	11	ND	ND	Silber et al. 2015 [11]

*SC* sub-cutaneous *ND* none determined

In total, 210 patients underwent a single transplantation procedure whereas 27 patients received second transplantation as well. A second transplantation procedure was performed either because of recurrence of the disease or because of short-term function of the transplanted tissue.

### Primary outcomes

A summary of the initial data is presented in Table 2. Return to menstruation as one of the primary outcomes was reported to take place between 2 months [26, 28, 31, 35, 40] to 1 year after transplantation [38]. The onset of ovarian activity and hormonal function at the closest time after transplantation was 1 week; estradiol and FSH levels showed an increase and decrease patterns, respectively [41]. Some of the recipients, however, did not respond to transplantation [26, 28, 34, 36, 40]. For example, in Dittrich et al. study, from 20 transplant recipients just one patient showed no activity after transplantation [17], whereas in one study about 50 percent of the patients exhibited ovarian function restoration after 12 months [28]. After second transplantation, ovarian function improved at 2–4 months post-transplantation and sustained for 9–84 months [42]. The ovarian function return varied in different studies between 3 and 6 months in the single frozen transplants [31]. In a 28 years old woman diagnosed with cervical cancer, however, ovarian function retrieval was observed 84 months after the second transplant. In this patient endocrine activity was observed even 7 years after transplantation [31]. Similarly, Silber et al. reported functional ovaries even after 8 years in two women who underwent fresh ovarian transplantation after the diagnosis of POF [11].

The evaluation of hormonal function after transplantation indicated that the level of FSH declined to less than 20 IU/l in 115 subjects while E2 levels had increased to above 100 pg/ml in 41 patients. These findings illustrate the success of transplantation. AMH levels, however, did not change in these cases, except for one study [11] in which the levels of AMH had increased after transplantation while the levels of FSH initially decreased, and then returned back to the normal levels [11]. In another study, a gradual elevation of AMH levels was observed during follow-up examination [37]. The inhibin level was assessed in various patients where it showed an increasing pattern [29, 39].

Dietrich et al. reported that the development of transplanted ovarian tissue in the pelvic wall led to the typical neo-ovarian regeneration [17]. The results from ovarian transplantation at different sites illustrated that the peritoneal cavity with a decent blood supply is a putative location for transplantation purposes in which a higher ratio of follicular development as well as higher number of antral follicles was observed [43].

Evaluation of follicular growth post-transplantation has been performed in some of the studies using ultrasonography, abdominal doppler, or vaginal echography, either for one or both ovaries [41]. Callejo et al. using ultrasonography and doppler identified the successful growth of follicles (one follicle with a 16 mm diameter) with surrounding blood flow in ovarian tissue transplanted to the abdominal rectus muscle [26]. In this line, Muller et al. confirmed the follicular growth (17 to 18 mm in diameter) using sonography, and subsequently ovulation induction was performed. Following a natural conception and a successful pregnancy, a healthy baby was born [18]. In another trial, dominant follicles were detected in ovaries transplanted to the peritoneum [39].

### Secondary outcomes

From the total number of 210 subjects who received the transplants, some failed to exhibit the desired secondary outcomes, out of which, several patients (age range 49–46 years) underwent bilateral hysterectomy and adnexectomy in order to prevent the symptoms of menopause [26]. Some patients did not attempt to maintain fertility [27, 31] as well as a single case of bilateral agenesis of fallopian tubes [35]. Out of remaining cases, 84 spontaneous and 36 IVF pregnancies, 80 live births, 22 abortions and 1 ectopic pregnancy were obtained (Table 3).

**Table 3:** Summary of secondary outcomes from selected studies

Total number patients	Type of tissue (cryopreservation/fresh)	Pregnancy	Spontaneous pregnancy	IVF pregnancy	Live birth	Abortion	(Author, year)
1	Cryo	–	–	–	–	–	Dittrich et al. 2008 [15]
1	Cryo	1	1	–	1	–	Dittrich et al. 2012 [16]
20	Cryo	7	6	1	4	1	Dittrich et al. 2015 [17]
1	Cryo	1	1	–	1	–	Müller et al. 2012 [18]
1	Cryo	–	–	–	–	–	Wølner-Hanssen et al. 2005 [19]
1	Cryo	1	1	1	–	–	Burmeister et al. 2013 [20]
1	Cryo	1	1	–	1	–	Revelli et al. 2013 [21]
1	Cryo	1	–	1	–	–	Kiseleva et al. 2015 [22]
1	Cryo	1	1	–	1	–	Demeestere et al. 2015 [10]
1	Cryo	2	1	1	2	–	Ernst et al. 2010 [23]
1	Cryo	4	1	–	3	1	Oktay et al. 2011 [24]
47	Cryo	1	1	–	1	–	Biasin et al. 2015 [25]
14	1 cryo, 3 fresh	–	–	–	–	–	Callejo et al. 2001 [26]
3	Fresh	–	–	–	–	–	Donnez et al. 2010 [27]
3	Cryo	–	–	–	–	–	Fabbri et al. 2014 [28]
10	7 cryo, 3 fresh	2	2	–	–	–	Janse et al. 2011 [29]
41	Cryo	28	13	15	13	3	Jensen et al. 2015 [30]
5	Cryo	–	–	–	–	–	Kim et al. 2012 [31]
56	Cryo	1	–	1	1	–	Meirow et al. 2007 [32]
1	Cryo	–	–	–	–	–	Fabbri et al. 2018 [33]
20	Cryo	16	7	9	10	3	Meirow et al. 2016 [34]
25	11 cryo, 11 fresh	21	19	2	17	7	Silber et al. 2010 [35]
47	Cryo	3	–	3	2	1	Suzuki et al. 2015 [36]
164	Cryo	2	1	1	2	–	Tanbo et al. 2015 [37]
399	Cryo	10	10	–	9	–	Beckmann et al. 2017 [38]
1	Cryo	1	1	–	–	1	Demeestere et al. 2006 [39]
225	Cryo	5	5	–	2	1	Imbert et al. 2014 [40]
1	Cryo	–	–	–	–	–	Povoa et al. 2016 [41]
22	11 cryo, 11 fresh	13	13	–	9	4	Silber et al. 2015 [11]

*Cryo* cryopreserved sample, – null

Of 29 recipients of fresh ovarian tissue transplantation, 26 subjects confirmed to be pregnant out of which 19 live births were reported with one confirmed pregnancy ongoing (20th week) at the end of the study. Of 181 frozen tissue recipients, 96 pregnancies and 86 live births were reported, with 8 continuing pregnancies at the end of the study.

The youngest worldwide reported case of ovarian tissue preservation and transplantation before puberty was a 9-year-old girl diagnosed with beta-thalassemia. In this specific patient, ovarian tissue was transplanted 14 years after cryopreservation and the normal ovarian function and pregnancy was confirmed following IVF [44]. In a similar case, a successful spontaneous pregnancy followed by live birth was reported for a patient who received ovarian tissue 10 years after removal at the age of 13 [10].

**Fig. 3:**
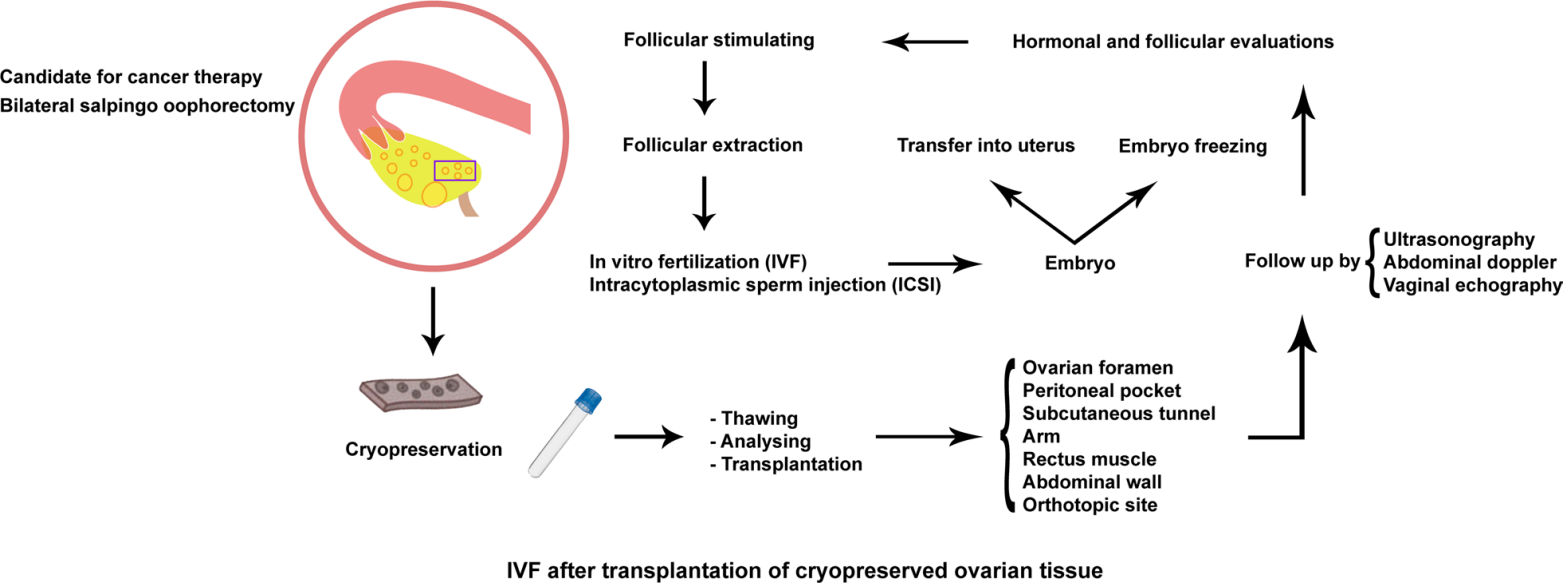
Overall conception of the IVF technique after transplantation of cryopreserved ovarian tissue

Considering other secondary outcomes, 11 embryos were obtained as a result of IVF cycles [23, 31, 40]. The summarized illustration of cryopreservation, transplantation, and IVF is shown in Fig. 3.

Regarding post-transplantation complications, among the reported abortions, one case has been diagnosed with chromosome 10 tetrasomy resulting in abortion at week 7 of pregnancy [39]. Seventeen cases of spontaneous abortion were described [11, 17, 33–35], and abortion was induced in 5 cases due to medical or personal issues (Table 3).

## Discussion

### The difference between fresh and frozen tissue transplantation

The selected papers described 181 patients that received frozen and 29 patients received fresh tissues; in 4 studies, patients received both fresh and frozen ovarian tissue [11, 26, 29, 35]. According to most studies, there was no significant difference regarding the function of the grafted ovaries whether fresh or frozen. In both fresh and frozen tissue recipients, ovarian activity was rejuvenated after 3–4 months [26]. In Janse et al. study, first menstruation in both fresh and frozen tissue recipients occurred after an average of 4.7 months [29]. In another study, no statistically significant differences were achieved related to FSH levels of the fresh and frozen tissue groups [35]. In a study by Silber et al. 4.5 months after the transplant, all recipients, both fresh and frozen, reached normal hormonal levels, exhibited ovulation and returned to a menstrual cycle within 130 days post-transplantation and the performance of fresh and frozen tissues was similar in both groups at least until 2 years after transplantation [11].

Our investigations illustrate the restoration of reproductive and endocrine function in most of the patients following ovarian tissue transplantation. In general, over 78% of the patients who received fresh or frozen ovarian tissue showed ovarian function restoration and in over 58% of patients, pregnancies occurred. Overall, 65% of live births were achieved in fresh tissue and 45% in frozen tissue recipients.

The efficacy of fresh and frozen transplanted tissues from identical twin sisters or HLA-matched sisters was reported for more than 2 years, and normal hormone levels and successful ovulation was observed 4–5 months after transplantation in all subjects. Serum levels of FSH in the patients showed a decline 3 days after transplantation and reached normal levels within 150 days after transplantation [11]. These observations clearly indicate the success of ovarian tissue transplantation.

In a study to examine the longevity of the cryopreserved tissues, ovarian pieces obtained from a 27-year-old woman were transplanted into Severe Combined Immune Deficiency (SCID) mice and monitored for 22 weeks. The results confirmed by morphology and proliferating cell nuclear antigen (PCNA) staining, indicated that cryopreservation of ovarian tissue maintains enough functionality and development of follicles, especially primordial ones post-freezing and even following transplantation of ovarian tissue [45].

Follicular count at the time of preservation is another important factor influencing ovarian tissue transplantation outcome. Biasin et al. confirmed that the functionality of ovarian tissue transplants with enough follicle counts was continued for almost 7 years with an average of 4–5 years [25]. In line with these findings, Silber reported that the duration of the ovarian function is directly linked to the ovarian reserve (primordial and antral follicle count) at the time of preservation [11]. These findings clearly illustrate that ovarian preservation at early ages with a high follicular reserve will increase the probability of success after transplantation. Indeed ovarian tissue preservation is believed to be the foremost clinical procedure for saving fertility in patients with pre-pubertal cancer, in which egg or embryo freezing is not possible [25].

Various anatomical sites have been practiced for ovarian tissue transplantation, and so far no significant differences in the outcomes such as follicular growth or restoration of ovarian function, were reported [39]. This is particularly important for patients who have lost their uterus and/or ovaries and were assisted to restore their endocrine functions [26]. In combination with IVF, fertility of the patient can be restored even if the ovarian tissue is not close to the oviduct [41]. On the other hand, Demeestere et al. described that orthotopic transplantation of ovarian tissue could provide a better environment, improved angiogenesis and blood supply required for the restoration of ovarian function [39]. The ovarian medulla has also been proposed as a good site for transplantation with high potential of angiogenesis [46].

Evaluation of the ovaries for possible malignancies before transplantation is a matter of utmost concern. A study done by Shaw et al. showed that transplantation of ovarian tissues from mice diagnosed with lymphoma into healthy ones caused over 90% of contamination in the recipients. Analyzing the absence or presence of malignant cells in the tissues considered in this study before tissue transplantation revealed no complications in the ovarian tissue preservation and transplantation safety [47].

### Cryopreservation procedure: slow freezing or vitrification?

Ovarian tissue cryopreservation is offered to preserve fertility and reproductive performance in women who are at high risk of POF and women who are subjected to cancer therapy in which oocyte or embryo preservation is not considered [8]. Vitrification and slow freezing are the general approaches proposed for cryopreservation of the ovary [13].

Silber et al. demonstrated no significant differences between fresh and frozen tissue transplantation (slow freezing method) groups in reduction of FSH to the basal levels and return of menstruation [11]. Similarly, in the study conducted by Klocke et al. comparing the quality of follicles immediately after freezing–thawing/vitrification–warming ovarian tissue, no significant differences between the two techniques were noticed [12]. In another study, comparison between transplantation of fresh tissue, vitrification, and slow-freezing tissue was performed. The outcome revealed that slow freezing technique caused low tissue survival as well as low follicle counts, probably due to the lyse of the stromal cells and nuclei density between bundles of extracellular fibers. However, no significant differences between fresh and vitrification groups were observed [35]. In Fabbri’s study, approximately 30% of the follicles in the slow freezing group showed signs of degeneration, probably due to osmotic stress [28]. In general, reports have shown that the ratio of healthy follicles and primordial follicle density with the vitrification method is higher than in with slow freezing technique [13]. Due to the high survival and lower level of follicular degeneration [36], vitrification is suggested as the most effective procedure for reserving and freezing ovarian tissue.

### Alternatives for ovarian transplantation

Various fertility preservation options are being offered for patients based on their health conditions. To determine the most appropriate choice, the status of the patient is critically evaluated by the physician [6]. Several options are available for fertility preservation such as egg or embryo cryopreservation, in vitro oocyte/follicle maturation and ovarian transposition or oophoropexy for radiation shielding [6].

Embryo and egg cryopreservation have certain limitations, such as the control of ovarian stimulation and, the time required in the ovulation stimulation process when for instance the cancer treatment has to start immediately after diagnosis [8].

In cases of hormone-sensitive malignancies such as several types of breast cancer, ovarian stimulation protocols different from the traditional protocols are required to superovulate and collect the oocytes. However, in young patients before puberty, in which ovarian stimulation protocols are not considered, ovarian tissue preservation is the only possible approach [8].

According to results from various investigations, cryopreservation of ovarian tissue before gonadotoxic treatments for women undergoing chemotherapy or radiotherapy is one of the available approaches to maintain reproductive capacity. This technique is an effective method for reserving and retaining thousands of follicles in the early stages of cancer diagnosis [8]. Preservation of the oocytes, embryos [48] and saving all or some part of the ovaries are steps that have been taken so far [7].

## Conclusion

After getting successful outcomes following ovarian tissue transplantation, researchers have tried to implement new technologies for transplantation surgery, aiming to use the least invasive methods. Hence, using the techniques with the lowest surgical invasion such as robot-assisted laparoscopic surgery, present new approaches for ovarian tissue transplantation aiming to reduce probable adverse effects [49]. Considering the results of our investigation, the ovarian tissue cryopreservation and transplantation technique is an applied and developed method for increasing the quality of life for women who are about to lose their fertility, patients diagnosed with premature ovarian failure (POF), and/or women undergoing cancer treatments. According to the results of studies, orthotopic transplantation has been shown to be the most effective method for resuscitation of endocrine function and restoration of fertility.

## Data Availability

Not applicable.

## Abbreviations

AMH: anti Mullerian hormone
FSH: follicle stimulating hormone
IVF: in-vitro fertilization
LH: luteinizing hormone
PRISMA: Preferred Reporting Items for Systematic Reviews
POF: premature ovarian failure
PCNA: proliferating cell nuclear antigen
